# Relationship between depression, anxiety, stress, and health-related quality of life in adults with and without chronic diseases: A cross-sectional study

**DOI:** 10.1097/MD.0000000000036967

**Published:** 2024-01-12

**Authors:** Younghui Hwang, Jihyun Oh

**Affiliations:** a Department of Nursing Science, College of Medicine, Chungbuk National University, Cheongju, Korea; b Department of Nursing, College of Nursing and Health, Kongju National University, Kongju, South Korea.

**Keywords:** anxiety, depression, health-related quality of life, stress

## Abstract

Health-related quality of life (HRQoL) in patients with chronic diseases is an important tool to measure patient-reported health outcomes and evaluate the usefulness of treatment, management, and rehabilitation programs. Patients with chronic diseases are more likely than those without to experience psychological problems such as depression, anxiety, and stress, all of which can affect HRQoL. This study evaluated the impact of psychological problems such as depression, anxiety, and stress on HRQoL in people with and without chronic diseases in South Korea. The study’s descriptive survey included 501 participants (191 with and 310 without chronic diseases). Data were collected using structured questionnaires between April and May 2021. The general characteristics, DASS-21, and HRQOL of this study were analyzed using descriptive statistics. Differences in DASS-21 and HRQoL based on general characteristics were analyzed using *t* tests and ANOVA. The study analyzed the factors influencing the participants’ HRQoL using stepwise multiple regression analysis with SPSS Win 27.0. HRQoL was generally lower for patients with chronic diseases than for patients without. In patients with chronic diseases, the major variables affecting HRQoL were depression (β = −0.244, *t* = −3.582, *P* < .001), exercise (β = 0.201, *t* = 2.927, *P* = .004), and economic status (β = −0.150, *t* = −2.184, *P* = .030), of which depression was the most influential. These variables explained 12.5% of the variance in the regression model for total HRQoL. These results emphasize the need to explore intervention measures that can reduce depression in patients with chronic diseases and anxiety in patients without chronic diseases to improve their HRQoL. In addition, national efforts are needed to provide economic support, as economic status is an influential factor in HRQoL regardless of the presence of chronic disease. The study’s limitations include the fact that neither did it consider disease severity among chronically ill patients nor did it examine all the variables affecting HRQoL.

## 1. Introduction

Patient-reported health outcomes are essential for helping healthcare providers comprehend the holistic burden of diseases, and health-related quality of life (HRQoL) is one method of measuring patient-reported health outcomes.^[1]^ HRQoL is a subjective concept that encompasses various dimensions of health,^[2]^ including how well people are able to function in their daily lives and how they perceive the physical, mental, and social dimensions of their lives.^[3]^ HRQoL can be an important tool for evaluating the usefulness of treatment, management, and rehabilitation programs for patients with chronic diseases^[4]^; moreover, research on HRQoL is gradually increasing.^[5]^

Finding ways to improve HRQoL requires identifying the variables affecting HRQoL and the manner in which they affect it. Various characteristics can impact HRQoL, such as demographics and physical and mental health status.^[6]^ Patients with chronic diseases such as diabetes, renal failure, and cervical compressive myelopathy may experience psychological problems such as depression, anxiety, and stress owing to symptoms related to the disease, frequent hospital visits, and a reduced role within the family or workplace.^[7–10]^ Depression usually presents as persistent sadness, lack of interest, and poor concentration and can dramatically affect a person’s ability to function and live a rewarding life.^[11,12]^ Moreover, depression can lead to unhealthy behaviors such as smoking, physical inactivity, and noncompliance with medication.^[11,13–15]^ These symptoms of depression and unhealthy behaviors can negatively affect HRQoL. Studies have reported that psychological factors such as anxiety and stress as well as depression have negative relationships with HRQoL in patients with chronic diseases.^[7–11]^ Thus, patients with a chronic disease are more likely to have psychological problems than those without a disease. In addition, the HRQoL effects of psychological problems on patients with a chronic disease will likely differ from their effects on those without a chronic disease. However, research on this issue is insufficient.

Amid the growing number of patients with multimorbidity^[16]^ (2 or more chronic illness at the same time),^[17,18]^it is essential to examine how the effects of various diseases impact patients’ overall health levels and HRQoL.^[19]^ It may be more beneficial to focus on various diseases or multimorbidity than on specific diseases when developing interventions for improving HRQoL at the population level.^[18]^ It may thus be useful to investigate how psychological problems affect HRQoL in people with chronic diseases as a whole, while also considering patients without chronic diseases to draw comparable outcomes.

Most studies have focused on identifying the impact of psychological problems on the HRQoL of patients with specific diseases.^[7–9]^ Therefore, this study investigated the effects of psychological problems such as depression, anxiety, and stress on HRQoL in people with and without chronic diseases. This study provides the basic data necessary for developing interventions designed to improve HRQoL in patients with various chronic diseases.

## 2. Methods

### 2.1. Study design and sample size

This study had a cross-sectional design. The study participants were adults aged ≥ 20 years residing in Seoul and Gyeonggi-do, South Korea. The study involved 501 participants. However, after 9 incomplete responses were excluded, the final sample consisted of 501 individuals. Among these, 310 had no diagnosed chronic diseases, whereas 191 had at least one diagnosed chronic condition.

### 2.2. Instruments

#### 2.2.1. General characteristics.

The general characteristics of the participants with and without chronic diseases were analyzed using a questionnaire that included questions on their age, gender, marital status, economic status, education level, religion, exercise, smoking, drinking, and type of chronic disease.

#### 2.2.2. Depression Anxiety Stress Scale-21.

The Depression Anxiety Stress Scale (DASS-21) is a 21-item self-report instrument that assesses 3 psychological constructs: depression, anxiety, and stress.^[20]^ Each subscale consists of 7 items. DASS-21 measures the psychological stress experienced by an individual over the previous week. An example of a depression item is “I couldn’t seem to experience any positive feeling at all.” An example of the 7 anxiety items is “I felt I was close to panic.” An example of the 7 stress items is “I found myself getting agitated.” The scores range from 0 to 3, where 0 indicates “not applicable to me at all” and 3 indicates “very much applicable to me, most of the time.” Higher scores indicate higher levels of psychological distress. The original DASS-21 instrument reported high reliability, with Cronbach’s α coefficients of 0.91, 0.84, and 0.90 for depression, anxiety, and stress, respectively. In this study, the DASS-21 Cronbach’s α coefficients for depression, anxiety, and stress were 0.86, 0.89, and 0.86, respectively, indicating high reliability.

#### 2.2.3. World Health Organization Quality of Life questionnaire.

The World Health Organization Quality of Life (WHOQOL-BREF)^[21]^ questionnaire is a brief version of the WHOQOL-100 used to measure HRQoL. This study used the Korean version of the WHOQOL-BREF-short version, which was translated into Korean and standardized by Min et al^[22]^ The WHOQOL-BREF consists of 26 questions divided into 2 questions on overall quality of life (QoL) and general health, 7 questions on physical health, 6 questions on psychological health, 3 questions on social relationships, and 8 questions on environmental domains. The participants were asked to indicate how satisfied they were with each of these domains in the past 4 weeks, with each item scored on a 5-point scale ranging from 1 (very poor/very dissatisfied/never/none) to 5 (very good/very satisfied/always/extremely). All domain scores were converted from 0 to 100, with higher scores indicating a higher HRQoL. The reliability of the tool at the time of development was Cronbach’s α = .90, while its reliability in this study was Cronbach’s α = .93.

### 2.3. Data collection

Ethical approval was obtained from the university’s Institutional Review Board prior to data collection. This study was conducted between April 22 and May 2, 2021, and involved adult men and women aged 20 years or older living in Seoul and Gyeonggi-do. The selection criteria for research participants were as follows: the participants were 20 years or older, had no communication impairments, had a clear consciousness, understood the purpose of the research, and provided consent to participate. The participants were also required to read and comprehend Korean. The study excluded all those who could not read or understand Korean or were unable to communicate. Those who agreed to participate in the survey filled out the consent form and completed the survey using a Google Forms link. Researchers provided detailed explanations about the purpose and methods of the study to the participants in person. Only those participants who agreed to participate signed the consent form, after which they were provided with the Google Forms link for the survey. Surveys were distributed to 510 adults aged 20 years and older. After 9 incomplete responses were excluded, data analysis was conducted on the responses of 501 participants.

### 2.4. Ethical considerations

The Research Ethics Committee of Daejeon University approved the study protocol (no. 1040647-202006-HR-011-03). All participants voluntarily consented to participate after receiving a face-to-face explanation of the study details from the researchers. This study adhered to the principles outlined in the Declaration of Helsinki.

### 2.5. Statistical analysis

The data collected in this study were analyzed in detail using SPSS 27.0 (IBM Corp., Armonk, NY. During the analysis of the data in this study, there were no missing data, excluding insufficient responses. To assess the normality of the research variables, the Shapiro–Wilk tests, skewness, and kurtosis were employed. The results indicate that the skewness ranges from −0.206 to 1.180, and the kurtosis ranges from −0.616 to 1.317. At a significance level of 0.05, the skewness is within ± 1.96, and the kurtosis is within an absolute value of 7, satisfying the assumption of normal distribution. Additionally, the Shapiro–Wilk test yielded a range between 0.056 and 0.091, confirming the normality of the data for this study.^[23]^

The general characteristics of participants with and without chronic diseases were analyzed using frequencies and percentages, and the differences between the 2 groups were analyzed using the chi-square test. Differences in DASS-21 and HRQoL according to general characteristics were analyzed using *t* tests and analysis of variance (ANOVA), and post hoc analyses were performed using the Scheffe test. Independent *t* tests were performed to compare DASS-21 and HRQoL between those with and without chronic diseases. The correlation between DASS-21 and HRQoL was analyzed using Pearson’s correlation coefficient. To identify the factors affecting the participants’ HRQoL, stepwise multiple regression was used.

## 3. Results

### 3.1. Participant characteristics

The participants were categorized into 2 groups: those with and those without chronic diseases. The average age of the participants without chronic diseases was 37.45 (8.28) years, and that of those with chronic diseases was 42.51 (10.11) years. A comparison of general characteristics between the 2 groups is shown in Table 1. Cross-analysis between the 2 groups categorized by age group (χ^2^ = 27.021, *P* < .001) and physical exercise showed significant differences (χ^2^ = 6.046, *P* = .014).

**Table 1:** Participant characteristics (N = 501).

Categories	No chronic disease (N = 310)	Chronic disease (N = 191)	*χ^2^*	*P*
	n (%)	n (%)		
Age	37.45 (8.28) (20–58)	42.51 (10.11) (20–73)		
20–29	55 (17.7)	21 (11.0)	27.021	<.001
30–39	122 (39.4)	49 (25.7)		
40–49	109 (35.2)	82 (42.9)		
≥50	24 (7.7)	39 (20.4)		
Sex				
Male	96 (31.0)	61 (31.9)	0.052	.820
Female	214 (69.0)	130 (68.1)		
Marital status				
Single	115 (37.1)	58 (30.4)	6.738	.304
Married	188 (60.6)	121 (63.4)		
Divorced/widowed	7 (2.3)	12 (6.3)		
Economic status				
High	19 (6.1)	11 (5.8)	0.030	.985
Middle	234 (75.5)	145 (75.9)		
Low	57 (18.4)	35 (18.3)		
Educational level				
≤High school	49 (15.8)	36 (18.8)	0.776	.378
≥College	261 (84.2)	155 (81.2)		
Religion				
Yes	153 (49.4)	105 (55.0)	1.494	.222
No	157 (50.6)	86 (45.0)		
Exercise				
Yes	137 (44.2)	106 (55.5)	6.046	.014
No	173 (55.8)	85 (44.5)		
Smoking status				
Yes	50 (16.1)	23 (12.0)	1.586	.208
No	260 (83.9)	168 (88.0)		
Alcohol consumption				
Yes	182 (58.7)	110 (57.6)	0.061	.805
No	128 (41.3)	81 (42.4)		
Type of chronic disease*				
High blood pressure		33 (15.9)		
High cholesterol		20 (9.6)		
Diabetes		9 (4.7)		
Musculoskeletal disease		41 (19.7)		
Thyroid disease		14 (6.7)		
Gastrointestinal disease		27 (13.0)		
Cardiac disease		8 (3.8)		
Tumor and Cancer		7 (3.4)		
Bronchiectasis		3 (1.4)		
Bladder disorders		3 (1.4)		
Other chronic disease		38 (18.3)		

* Multiple response.

Of the 501 participants, 310 (61.9%) did not have a chronic disease. This group’s age distribution was as follows: 17.7% were in their 20s, 39.4% were in their 30s, 35.2% were in their 40s, and 7.7% were aged 50 years or older. Regarding sex, 69.0% of the participants were women. Regarding marital status, 60.6% of the participants were married. Based on the self-reported questionnaires, the economic status of the participants was divided into 3 levels: high, medium, and low. Those who reported their economic status as “medium” accounted for 75.5% of the participants. Moreover, 84.2% of the participants had a college degree or higher, 50.6% were not religious, 55.8% did not exercise, 83.9% did not smoke, and more than half (58.7%) consumed alcohol.

Among the participants, 191 had a chronic disease. Of these 191 individuals, 11.0% were in their 20s, 25.7% were in their 30s, 42.9% were in their 40s, and 20.4% were 50 years or older; 68.1% were women, 31.9% were men, and 63.4% were married. Regarding economic status, 75.9% of the participants perceived themselves as belonging to the “middle” class. In addition, 81.2% had a college degree or higher, while 55.0% identified with a religion, and 55.5% engaged in regular physical activity. nonsmokers comprised 88.0% of the group, and 57.6% consumed alcohol. We analyzed the frequency of specific chronic diseases in patients diagnosed with chronic conditions, finding that 15.9% had hypertension, 9.6% had hypercholesterolemia, and 4.7% had diabetes mellitus. However, musculoskeletal disorders (such as disc problems and arthritis) were the most prevalent conditions, accounting for 19.7% of the cases. Participants with thyroid conditions made up 6.7% of the group, those with gastrointestinal diseases comprised 13.0%, and 18.3% had other ailments.

### 3.2. Differences in DASS-21 scores based on general characteristics between participants with and without chronic diseases

This study analyzed the differences in DASS-21 scores based on the general characteristics of the 310 participants without chronic diseases (see Table 2). Analysis of the differences in DASS-21 scores based on economic status revealed significant differences in DASS-21 depression (*F* = 5.205, *P* = .006), anxiety (*F* = 4.956, *P* = .008), and stress scores (*F* = 5.205, *P* = .006). A post hoc analysis using the Scheffe test found that the DASS-21 depression score for participants with a “low” economic status was 6.77 (4.31), which was significantly higher than the score of 4.85 (4.02) for participants with a “medium” economic status. The DASS-21 anxiety score for participants with a “low” economic status was 4.98 (4.34), which was significantly higher than the score of 3.32 (4.57) for participants with a “medium” economic status. Similarly, the stress score of 6.77 (4.31) for those reporting “low” economic status was significantly higher than the stress score of 4.85 (4.02) for those reporting “middle” economic status.

**Table 2:** Differences in DASS-21 based on general characteristics among participants with and without chronic disease (N = 501).

Variables	DASS-21 depression	*F*/*t*	DASS-21 anxiety	*F*/*t*	DASS-21 stress	*F*/*t*
	Mean (SD)	*P*	Mean (SD)	*P*	Mean (SD)	*P*
No chronic disease (N = 310)						
Age						
20–29	5.56 (4.64)	1.812 (.145)	3.72 (4.23)	1.783 (.150)	5.56 (4.64)	1.812 (.145)
30–39	5.77 (4.31)		4.24 (4.34)		5.77 (4.31)	
40^–^49	4.53 (3.86)		3.06 (3.27)		4.53 (3.86)	
≥50	5.58 (4.27)		4.04 (4.50)		5.58 (4.27)	
Sex						
Male	4.81 (4.29)	−1.313 (.190)	3.67 (3.91)	−.135 (.892)	4.81 (4.29)	−1.313 (.190)
Female	5.49 (4.20)		3.74 (3.98)		5.49 (4.20)	
Marital status						
Single	4.83 (4.15)	1.456 (.235)	3.52 (3.69)	.718 (.488)	4.83 (4.15)	1.456 (.235)
Married	5.49 (4.22)		3.78 (4.03)		5.49 (4.22)	
Divorced/widowed	7.00 (5.59)		5.28 (6.10)		7.00 (5.59)	
Economic status						
High^a^	6.10 (5.51)	5.205 (.006) b < c	4.84 (6.02)	4.956 (.008) b < c	6.10 (5.51)	5.205 (.006) b < c
Middle^b^	4.85 (4.02)		3.32 (3.57)		4.85 (4.02)	
Low^c^	6.77 (4.31)		4.98 (4.34)		6.77 (4.31)	
Educational level						
≤High school	4.89 (3.78)	−.694 (.488)	3.85 (3.72)	.259 (.796)	4.89 (3.78)	−.694 (.488)
≥College	5.35 (4.32)		3.69 (4.00)		5.35 (4.32)	
Religion						
Yes	5.60 (4.31)	1.330 (.185)	3.70 (3.92)	−.073 (.942)	5.60 (4.31)	1.330 (.185)
No	4.96 (4.15)		3.73 (4.00)		4.96 (4.15)	
Exercise						
Yes	5.04 (4.11)	−.887 (.376)	3.59 (4.05)	−.519 (.604)	5.04 (4.11)	−.887 (.376)
No	5.47 (4.33)		3.82 (3.88)		5.47 (4.33)	
Smoking						
Yes	5.40 (4.17)	.211 (.833)	4.32 (4.12)	1.166 (.244)	5.40 (4.17)	.211 (.833)
No	5.26 (4.25)		3.60 (3.92)		5.26 (4.25)	
Alcohol						
Yes	5.34 (4.18)	.281 (.779)	3.65 (3.87)	−.364 (.716)	5.34 (4.18)	.281 (.779)
No	5.20 (4.33)		3.82 (4.08)		5.20 (4.33)	
Chronic disease (N = 191)						
Age						
20–29	6.29 (3.78)	1.812 (.145)	4.57 (3.78)	1.783 (.150)	6.29 (3.48)	1.812 (.145)
30–39	7.86 (4.48)		6.47 (4.80)		7.86 (4.48)	
40^–^49	6.01 (.3.91)		4.57 (3.72)		6.01 (3.91)	
≥50	5.62 (3.84)		3.95 (3.65)		5.62 3.84)	
Sex						
Male	6.42 (3.93)	−.019 (.985)	5.19 (4.02)	.611 (.542)	6.42(3.93)	−.019 (.985)
Female	6.43 (4.18)		4.80 (4.13)		6.43(4.18)	
Marital status						
Single	6.60 (4.06)	.121 (.886)	4.66 (3.58)	.391 (.677)	6.60 (4.06)	.121 (.886)
Married	6.40 (4.14)		5.12 (4.42)		6.40 (4.14)	
Divorced/widowed	6.00 (4.16)		4.33 (3.03)		6.00 (4.16)	
Economic status						
High	7.36 (4.57)	.470 (.626)	6.36 (4.61)	.772 (.463)	7.36 (4.57)	.470 (.626)
Middle	6.29 (4.05)		4.90 (4.16)		6.29 (4.05)	
Low	6.74 (6.74)		4.63 (3.65)		6.74 (4.21)	
Educational level						
≤High school	6.16 (3.76)	−.435 (.664)	4.63 (3.89)	−.476 (.635)	6.16 (3.76)	−.435 (.664)
≥College	6.49 (4.18)		5.00 (4.14)		6.49 (4.18)	
Religion						
Yes	6.58 (4.26)	.545 (.587)	5.06 (4.33)	.502 (.617)	6.58 (4.26)	.545 (.587)
No	6.25 (3.90)		4.76 (3.80)		6.25 (3.90)	
Exercise						
Yes	6.15 (4.01)	−.667 (.505)	4.75 (4.16)	−.667 (.505)	6.15 (4.01)	−1.069 (.287)
No	6.78 (4.19)		5.15 (4.01)		6.78 (4.19)	
Smoking						
Yes	7.69 (4.05)	1.580 (.116)	6.86 (4.07)	2.452 (.015)	7.69 (4.05)	1.580 (.116)
No	6.26 (4.08)		4.66 (4.03)		6.26 (4.08)	
Alcohol						
Yes	6.90 (4.19)	1.878 (.062)	5.41 (4.25)	1.927 (.056)	6.90 (4.19)	1.878 (.062)
No	5.79 (3.89)		4.27 (3.78)		5.79 (3.89)	

This study examined the differences in DASS-21 scores for depression, anxiety, and stress among the 191 participants with chronic diseases based on their general characteristics. We found that the anxiety of smokers was 6.86 (4.07), which was higher than that of nonsmokers, which was 4.66 (4.03) (*t* = 2.452, *P* = .015).

### 3.3. Differences in HRQoL based on general characteristics of participants with and without chronic diseases

In the group without chronic diseases, we analyzed the differences in HRQoL according to their general characteristics (see Table 3). Statistically significant differences were observed in overall QoL and general health (*F* = 14.037, *P* < .001), physical health (*F* = 9.019, *P* < .001), psychological health (*F* = 16.587, *P* < .001), social relationships (*F* = 10.761, *P* < .001), environmental domains (*F* = 25.163, *P* < .001), and total HRQoL (*F* = 21.223, *P* < .001), based on the economic status of the participants.

**Table 3:** Differences in WHOQOL-BREF based on general characteristics among participants with and without chronic disease (N = 501).

No chronic disease (N = 310)	Overall and general	*F/t*	Physical health	*F/t*	Psychological health	*F/t*	Social relationship	*F/t*	Environmental health	*F/t*	Total HRQoL	*F/t*
Variables	Mean (SD)	*P*	Mean (SD)	*P*	Mean (SD)	*P*	Mean (SD)	*P*	Mean (SD)	*P*	Mean (SD)	*P*
Age												
20–29	64.36 (17.61)	.894 (.444)	65.66 (13.38)	1.289 (.278)	63.63 (14.13)	.163 (.921)	60.72 (18.48)	.965 (.410)	60.77 (17.59)	.941 (.421)	63.03 (42.28)	.808 (.490)
30–39	68.11 (12.55)		67.40 (11.93)		64.69 (12.71)		63.38 (14.52)		63.40 (14.44)		65.40 (11.10)	
40^–^49	66.60 (13.62)		69.35 (11.69)		63.91 (11.72)		64.95 (13.71)		64.77 (12.94)		65.92 (10.29)	
≥59	66.66 (16.06)		68.80 (10.28)		65.13 (10.53)		63.05 (15.22)		62.81 (13.45)		65.29 (11.29)	
Sex												
Male	69.16 (12.86)	1.969 (.050)	69.82 (12.15)	1.903 (.058)	65.55 (10.88)	1.221 (.308)	64.79 (14.07)	1.056 (.308)	65.36 (13.75)	1.627 (.308)	66.94 (10.83)	1.842 (.308)
Female	65.74 (14.66)		67.02 (11.89)		63.69 (13.05)		62.83 (15.50)		62.47 (14.74)		64.35 (11.67)	
Marital status												
Single	66.52 (14.39)	.392 (.676)	68.17 (12.89)	.086 (.917)	63.88 (12.97)	.679 (.508)	61.79 (16.52)	1.114 (.330)	61.91 (15.85)	1.112 (.330)	64.45 (12.62)	.519 (.596)
Married	66.80 (14.12)		67.76 (11.63)		64.30 (12.20)		64.36 (14.20)		64.10 (13.54)		65.47 (10.78)	
Divorced/widowed	71.42 (14.63)		66.53 (8.03)		69.52 (9.51)		65.71 (12.42)		67.50 (15.13)		68.13 (9.75)	
Economic status												
High^a^	77.36 (12.40)	14.037 (<.001)c < b < a	71.42 (11.34)	9.019 (<.001)a,b > c	71.75 (10.44)	16.587 (<.001)a,b > c	70.17 (13.21)	10.761 (<.001)a,b > c	73.81 (13.13)	25.163 (<.001)a > b > c	72.90 (10.52)	21.223<.001a > b > c
Middle^b^	67.69 (13.12)		69.02 (12.01)		65.49 (12.02)		64.75 (14.78)		65.09 (13.57)		66.41 (10.68)	
Low^c^	59.64 (15.91)		62.05 (10.54)		56.72 (11.57)		55.78 (14.39)		52.80 (13.11)		57.40 (11.26)	
Educational level												
≤High school	60.20 (16.00)	−3.615 (<.001)	63.09 (13.30)	−3.087 (.002)	59.04 (13.86)	−3.252 (.001)	57.00 (19.00)	−3.306 (.001)	55.20 (17.23)	−4.429 (<.001)	58.91 (14.40)	−4.268 (<.001)
≥College	68.04 (13.51)		68.79 (11.57)		65.24 (11.92)		64.64 (13.94)		64.90 (13.39)		66.32 (10.45)	
Religion												
Yes	66.66 (15.17)	−.171 (.865)	68.21 (12.13)	.472 (.637)	64.50 (12.45)	.336 (.737)	63.57 (14.73)	.152 (.879)	63.66 (13.86)	.346 (.729)	65.32 (11.34)	.257 (.797)
No	66.94 (13.23)		67.57 (11.94)		64.03 (12.46)		63.31 (15.46)		63.08 (15.08)		64.98 (11.62)	
Exercise												
Yes	68.68 (12.82)	2.085 (.038)	69.71 (12.52)	2.402 (.017)	65.83 (12.11)	1.988 (.048)	62.72 (13.82)	−.743 (.458)	64.52 (14.02)	1.250 (.212)	66.29 (11.13)	1.566 (.118)
No	65.31 (15.07)		66.44 (11.43)		63.02 (12.58)		64.00 (16.02)		62.45 (14.81)		64.24 (11.67)	
Smoking												
Yes	65.40 (15.28)	−.764 (.445)	66.22 (11.64)	−1.067 (.287)	61.13 (10.66)	−1.955 (.051)	62.66 (14.93)	−.396 (.639)	62.00 (14.37)	−.730 (.466	63.47 (11.45)	−1.125 (.262)
No	67.07 (14.01)		68.20 (12.09)		64.87 (12.68)		63.58 (15.13)		63.63 (14.51)		65.47 (11.45)	
Alcohol												
Yes	66.09 (14.05)	−1.046 (.296)	67.78 (11.59)	−.179 (.858)	64.70 (11.95)	−.739 (.460)	63.40 (14.18)	−.048 (.962)	63.64 (13.92)	.390 (.697)	65.12 (10.99)	−.050 (.960)
No	67.81 (14.41)		68.03 (12.65)		63.64 (13.11)		63.48 (16.32)		62.98 (15.28)		65.19 (12.15)	
Chronic disease (N = 191)	Overall and general	F/t	Physical health	F/t	Psychological health	F/t	Social relationship	F/t	Environmental health	F/t	Total QOL	F/t
Variables	Mean (SD)	*P*	Mean (SD)	*P*	Mean (SD)	*P*	Mean (SD)	*P*	Mean (SD)	*P*	Mean (SD)	*P*
Age												
20–29	61.42 (13.52)	1.694 (.170)	67.48 (12.27)	1.309 (.273)	60.00 (14.10)	.275 (.843)	67.94 (15.29)	3.220 (.024)	66.19 (13.87)	.870 (.458)	64.61 (10.09)	1.080 (.359)
30–39	59.59 (15.67)		62.68 (11.76)		63.06 (13.33)		62.18 (13.01)		62.45 (14.80)		61.99 (11.18)	
40–49	64.76 (15.81)		66.62 (12.59)		62.52 (14.29)		68.29 (14.38)		65.24 (14.41)		65.49 (11.68)	
≥50	65.38 (10.72)		64.91 (11.83)		62.91 (12.10)		61.71 (13.04)		61.73 (14.40)		63.33 (10.49)	
Sex												
Male	63.44 (15.90)	.160 (.873)	66.08 (10.82)	.095 (.571)	62.56 (14.16)	.963 (.940)	66.66 (11.92)	.099 (.375)	65.45 (12.91)	.512 (.315)	64.84 (10.55)	.479 (.504)
Female	63.07 (14.18)		65.01 (12.85)		62.41 (13.27)		64.71 (15.04)		63.19 (15.09)		63.68 (11.46)	
Marital status												
Single	59.83 (17.01)	2.250 (.108)	65.91 (12.27)	.101 (.904)	60.69 (14.53)	.801 (.451)	64.48 (15.16)	.554 (.575)	62.72 (15.36)	.744 (.476)	62.73 (12.30)	.693 (.502)
Married	64.55 (13.29)		65.05 (12.05)		63.39 (13.11)		66.06 (13.74)		64.81 (14.23)		64.77 (10.71)	
Divorced/widowed	65.83 (15.05)		65.71 (14.62)		61.67 (12.83)		62.22 (13.13)		60.63 (11.83)		63.21 (10.17)	
Economic status												
High^a^	73.64 (18.04)	7.341 (.001)a,b.>c	70.39 (13.60)	1.597 (.205)	66.97 (12.86)	2.063 (.130)	66.67 (9.43)	.134 (.874)	65.91 (13.15)	2.120 (.123)	68.71 (11.77)	3.087 (.048)
Middle^b^	64.07 (13.77)		65.54 (12.40)		63.01 (13.60)		65.47 (14.86)		64.84 (14.91)		64.59 (11.31)	
Low^c^	56.29 (14.97)		63.02 (10.73)		58.76 (12.96)		64.38 (12.31)		59.43 (12.11)		60.38 (9.59)	
Educational level												
≤High school	62.77 (13.44)	−.188 (.851)	62.06 (12.29)	−1.804 (.073)	59.90 (11.99)	−1.259 (.210)	63.88 (15.23)	−.684 (.495)	60.6914.63)	−.490 (.138)	61.86 (10.86)	−1.306 (.193)
≥College	63.29 (15.03)		66.11 (12.13)		63.05 (13.82)		65.67 (13.88)		64.66 (14.33)		64.56 (11.21)	
Religion												
Yes	64.76 (13.94)	1.634 (.104)	64.54 (12.82)	−1.013 (.312)	63.33 (13.10)	.985 (.326)	65.01 (13.95)	−.350 (.727)	63.71 (14.70)	−.210 (.834)	64.27 (11.28)	.302 (.763)
No	61.27 (15.47)		66.34 (11.44)		61.39 (14.02)		65.73 (14.39)		64.15 (14.19)		63.78 (11.08)	
Exercise												
Yes	65.84 (13.86)	2.836 (.005)	67.08 (12.29)	2.211 (.028)	64.46 (13.75)	2.313 (.022)	68.05 (12.40)	3.025 (.003)	66.83 (13.71)	3.203 (.002)	66.45 (10.59)	3.417 (.001)
No	59.88 (15.15)		63.19 (11.85)		59.96 (12.88)		61.96 (15.42)		60.26 (15.46)		61.05 (11.20)	
Smoking												
Yes	61.73 (18.25)	−.504 (.615)	64.72 (12.44)	−.265 (.791)	61.01 (13.61)	−.546 (.586)	63.47 (14.05)	−.673 (.502)	62.71 (14.71)	−.423 (.673)	62.71 (11.63)	−.603 (.547)
No	63.39 (14.21)		65.44 (12.23)		62.65 (13.54)		65.59 (14.15)		64.07 (14.34)		64.23 (11.12)	
Alcohol												
Yes	62.81 (14.90)	−.410 (.682)	65.32 (11.64)	−.040 (.968)	61.51 (13.63)	−1.127 (.261)	67.09 (14.22)	2.013 (.046)	65.00 (13.99)	1.213 (.227)	64.34 (11.47)	.427 (.670)
No	63.70 (14.52)		65.32 (11.64)		63.74 (13.35)		62.96 (13.70)		62.43 (14.98)		63.64 (11.47)	

We also examined the differences in total HRQoL and each of its subdomains according to the participants’ educational levels. Significant differences were found across all domains, including overall QoL and general health (*t* = −3.615, *P* < .001), physical health (*t* = −3.087, *P* = .002), psychological health (*t* = −3.252, *P* = .001), social relationships (*t* = −3.306, *P* = .001), environmental domains (*t* = −4.429, *P* < .001), and total HRQoL (*t* = −4.268, *P* < .001). Participants with a college degree or higher had HRQoL scores significantly higher than those of participants with high school education or lower. Statistically significant differences emerged in relation to regular exercise in the domains of overall QoL and general, physical, and psychological health. Specifically, in terms of overall QoL and general health, participants who engaged in regular exercise reported scores of 68.68 (12.82), which were significantly higher than the scores of 65.31 (15.07) for those who did not engage in regular exercise. Within the physical health domain, those who exercised regularly scored 69.71 (12.52), significantly outperforming the score of 66.44 (11.43) of non-exercising participants. As for the psychological health domain, participants who exercised regularly scored 65.83 (12.11), which was statistically higher than the score of 63.02 (12.58) for those who did not engage in regular exercise.

The study also examined differences in HRQoL based on the general characteristics of the 191 participants with chronic diseases (see Table 3). A statistically significant difference was observed in the HRQoL subdomain, specifically in social relationships, based on age (*F* = 3.220, *P* < .024). Statistically significant differences were also observed in overall QoL and general health (*F* = 7.341, *P* = .001), as well as total HRQoL (*F* = 3.087, *P* = .048), based on perceived economic status. The study also found statistically significant differences in all subdomains of HRQoL as well as in overall QoL and general health based on physical exercise. Individuals who exercised regularly had significantly higher scores in all HRQoL domains than those who did not engage in regular exercise. This study also examined differences in HRQoL based on alcohol consumption and found a significant difference in social relationships (*t* = 2.013, *P* = .046). Participants who consumed alcohol had a social relationship score of 67.09 (14.22), which was statistically higher than the score of 62.96 (13.70) for participants who did not consume alcohol.

### 3.4. Differences in variables between the 2 groups

The research findings revealed a significant difference in DASS-21 depression scores between the group with chronic diseases and the group without (see Table 4). A statistically significant difference was observed in DASS-21 depression scores between the 2 groups (*t* = −3.274, *P* = .001). Specifically, the group without chronic diseases had a DASS-21 depression score of 4.12 (4.16), whereas the group with chronic diseases had a DASS-21 depression score of 5.40 (4.37). This finding indicates that individuals with a chronic disease have higher levels of depression. The study also found a significant difference in DASS-21 anxiety scores between the 2 groups (*t* = −3.279, *P* = .001). Specifically, the group without chronic diseases had a DASS-21 anxiety score of 3.72 (3.95), whereas the group with chronic diseases had a score of 4.93 (4.09), indicating higher anxiety levels in the latter group. Additionally, there was a notable difference in DASS-21 stress scores between the groups (*t* = −2.989, *P* = .003). The group without chronic diseases had a DASS-21 stress score of 5.28 (4.23), compared with a score of 6.63 (4.09) in the group with chronic diseases, suggesting elevated levels of stress in those with chronic conditions.

**Table 4:** Differences in variables between the 2 groups.

Variables	No chronic disease (N = 310)	Chronic disease (N = 191)	*t*	*P*
	Mean (SD)			
DASS-21 Depression	4.12 (4.16)	5.40 (4.37)	−3.274	.001
DASS-21 Anxiety	3.72 (3.95)	4.93 (4.09)	−3.279	.001
DASS-21 Stress	5.28 (4.23)	6.43 (4.09)	−2.989	.003
Overall QOL and general health	66.80 (14.20)	63.19 (14.71)	2.727	.007
Physical health	67.88 (12.02)	65.35 (12.22)	2.277	.023
Psychological health	64.26 (12.43)	62.46 (13.52)	1.528	.127
Social relationship	63.44 (15.08)	65.34 (14.12)	−1.402	.161
Environmental health	63.37 (14.48)	63.91 (14.43)	−.408	.684
Total HRQoL	65.15 (11.46)	64.05 (11.16)	1.056	.292

DASS-21 = Depression Anxiety Stress Scale-21, HRQoL = health-related quality of life, SD = standard deviation.

The study then compared between the group with chronic diseases and the group without in terms of various HRQoL subdomains. Significant differences were found only in overall QoL, general health, and physical health. Specifically, scores for overall QoL and general health averaged 66.80 (14.20) among participants without chronic diseases and 63.19 (14.71) among those with chronic conditions, indicating statistically lower levels in the latter group (*t* = 2.727, *P* = .023). Furthermore, the average physical health score was 67.88 (12.02) for those without chronic diseases and 65.35 (12.22) for those with chronic diseases, indicating significantly lower scores in the group with chronic diseases (*t* = 2.277, *P* = .023).

### 3.5. Correlation among variables

Table 5 shows the correlations between the study’s variables: DASS-21 depression, anxiety, stress, and HRQoL.

**Table 5:** Correlation coefficients among DASS-21 and WHOQOL-BREF.

Variables	DASS-21			WHOQOL-BREF					
	1	2	3	4	5	6	7	8	9
1.DASS-21 Depression	1								
2.DASS-21 Anxiety	0.847**	1							
3.DASS-21 Stress	1**	0.847**	1						
4.Overall and general	−0.255**	−0.199**	−0.225**	1					
5.Physical health	−0.374**	−0.365**	−0.374**	0.610**	1				
6.Psychological health	−0.248**	−0.239**	−0.248**	0.683**	0.660**	1			
7.Social relationship	−0.086	−0.089	−0.086	0.398**	0.497**	0.344**	1		
8.Environmental health	−0.213**	−0.191**	−0.213*	0.642**	0.703**	0.760**	0.751**	1	
9.Total HRQoL	−0.278**	−0.255**	−0.278**	0.807**	0.828**	0.825**	0.737**	0.936**	1

DASS-21 = Depression Anxiety Stress Scale-21, HRQoL = health-related quality of life, WHOQOL = World Health Organization Quality of Life.

*P < .05.

** *P* < .01.

### 3.6. Factors affecting HRQoL among participants

Table 6 presents the results of stepwise regression analysis conducted to examine the factors affecting HRQoL in participants with and without chronic diseases. Categorical variables such as economic status, education level, and exercise were included as dummy variables. Independent variables such as the DASS-21 scores for depression, anxiety, and stress were also included in the stepwise regression analysis. The variables were selected using a significance probability of 0.5. Based on the stepwise regression analysis model predicting the HRQoL of participants without chronic diseases, the significant variables were DASS-21 anxiety (β = −0.254, *t* = −4.990, *P* < .001), economic status (β = −0.291, *t* = −5.628, *P* < .001), and education level (β = −.179, *t* = 3.471, *P* < .001). These 3 major variables explained 20.7% of the variance in HRQoL for participants without chronic diseases. Among these variables, economic status was the most influential factor, followed by DASS-21 anxiety and educational level.

**Table 6:** Factors affecting WHOQOL-BREF among participants.

Variable	Non-diseased group (N = 300)			Diseased group (N = 191)		
	B (SE)	β	*t (P*)	B (SE)	β	*t (P*)
Constant	72.299 (4.360)		16.582 (<.001)	83.321 (4.023)		30.463 (<.001)
DADD-21 Depression				−0.665 (0.186)	−0.244	−3.582 (<.001)
DASS-21 Anxiety	−0.735 (0.147)	−0.254	−4.990 (<.001)			
Exercise				4.512 (1.541)	0.201	2.927 (.004)
Economic status	−6.949 (1.235)	−0.291	−5.628 (<.001)	−3.515 (1.610)	−0.150	−2.184 (.030)
Education level	5.616 (1.618)	0.179	3.471 (<.001)			
*R* ^2^	0.214			0.139		
Adj. *R*^2^	0.207			0.125		
*F*	27.843 (<0.001)			10.027 (<0.001)		

DASS-21 = Depression Anxiety Stress Scale-21, WHOQOL = World Health Organization Quality of Life.

Stepwise regression analysis was also conducted to analyze the important factors affecting HRQoL in the participants with chronic diseases. The regression model consisted of the statistically significant factors related to HRQoL. Table 6 presents the results. We analyzed variables related to changes in HRQoL. The results show that the major variables affecting HRQoL were depression (β = −0.244, *t* = −3.582, *P* < .001), exercise (β = 0.201, *t* = 2.927, *P* = .004), and economic status (β = −0.150, *t* = −2.184, *P* = .030). These 3 variables explained 12.5% of the variance in the regression model for total HRQoL. Among these 3 variables, depression had the greatest influence, followed by exercise and economic status.

## 4. Discussion

The main finding of this study was that depression was the factor most affecting HRQoL for patients with chronic diseases, followed by exercise and economic status, while economic status was the most influential factor affecting HRQoL for patients without chronic diseases, followed by anxiety and education level.

The finding that depression is the factor that most influenced HRQoL for patients with chronic disease is supported by the finding in previous studies that chronic disease patients with depression had lower QoL than patients without depression^[24]^ and that depression in chronic diseases (such as inflammatory bowel disease, diabetes, and cardiovascular disease) is associated with low HRQoL.^[25–27]^ Chronic disease patients are more likely to develop depression owing to disease-related symptoms, frequent hospital visits, and reduced roles in their families and workplaces^[7–10]^; depression can also reduce interest, decrease concentration, and lower daily functioning.^[11,12]^ In addition, depression can negatively affect health and HRQoL because it is linked to unhealthy or risky behaviors such as smoking, overeating, a sedentary lifestyle, and non-adherence to medication^[11,13–15]^ In other words, it can be assumed that depression has a considerable impact on health and HRQoL by affecting the overall health-related behavior of patients with chronic diseases. Further research is needed to reveal how health-related behaviors such as smoking, eating, physical activity, and medication adherence mediate the relationship between depression and HRQoL. Additionally, environmental factors such as exposure to endocrine disruptors are also considered to be a cause of depression.^[28]^ More research is needed on the relationship between depression, chronic diseases, environments, and HRQoL.

Depression is an important public health issue, and its incidence is gradually increasing worldwide,^[29]^particularly among people with chronic diseases.^[27]^ Incidence cases of depression worldwide increased almost 1.5 times, from 172 million in 1990 to 258 million in 2017.^[29]^ Research also shows that depression can be a key factor in the development of chronic diseases such as arthritis or hypertension.^[27]^ Depression is not only associated with lower HRQoL but also with increased mortality and greater health care costs.^[25–27]^ The findings of this study suggest that treating depression in patients with chronic diseases is as important as treating the disease itself. Therefore, to enhance the HRQoL of patients with chronic diseases, it is necessary to periodically check all patients with chronic diseases for depression, and healthcare providers must be aggressive in their efforts to reduce depression in patients with chronic diseases.^[26]^

Exercise was the next most-important factor affecting HRQoL in the group with chronic diseases. This finding is consistent with the results of previous studies suggesting that exercise programs can improve HRQoL in patients with hypertension,^[30]^ diabetes,^[31]^ chronic kidney disease,^[32]^ and inflammatory bowel conditions.^[33]^ Exercise in patients with hypertension or diabetes has been shown to improve hemodynamic profiles and cardiopulmonary fitness, such as body mass index, waist circumference, systolic blood pressure, and peak oxygen consumption, thereby enhancing HRQoL in terms of physical function, general health, vitality, and mental health.^[30,31]^ Moreover, exercise can be effective in reducing the occurrence of depression and its symptoms.^[34]^ Thus, it is necessary to encourage patients with chronic diseases to exercise in ways suitable to their condition.

Finally, economic status was found to affect HRQoL in patients with chronic diseases, which is consistent with the results of previous studies.^[4,6]^ The financial burden of medical expenses for chronic diseases is high,^[35]^ affecting patients’ HRQoL. Thus, providing financial support programs should be prioritized as a way to improve patients’ HRQoL.^[4]^

This study found that depression and exercise were factors that influenced HRQoL for patients with chronic diseases but not for those without chronic diseases. Interestingly, people without chronic diseases had lower depression levels and lower rates of regular exercise than patients with chronic diseases, which may be why depression and exercise did not have a significant impact on HRQoL in people without chronic diseases. In other words, patients without disease had a lower incidence of depression and exercise than patients with disease. Thus, it can be inferred that depression and exercise did not affect HRQoL. This result is similar to the research finding that unhealthy office workers showed greater improvements in HRQOL owing to exercise than healthy office workers did^[36]^ and that the severity of depression symptoms was found to be related to HRQoL.^[37]^ However, some studies have also shown that exercise and depression affect HRQoL in healthy adults^[38,39]^ and that the impact on HRQoL may vary depending on the type and method of exercise.^[40]^ Further research is needed on these issues.

Economic status was the factor that most strongly influenced the HRQoL of people without chronic diseases, while also impacting that of patients with chronic diseases. Among people without chronic diseases, educational level was the third most-influential factor on HRQoL. Considering that education level is a socioeconomic factor that reflects economic status,^[41,42]^ economic status may have a significant impact on HRQoL irrespective of the presence of chronic diseases.^[4,5]^ Since economic inequality can cause health inequality,^[42]^ national efforts to address it should continue.

Anxiety was the second most-significant factor affecting HRQoL in individuals without chronic diseases. Anxiety is associated with unhealthy behaviors such as reduced activity, insomnia, smoking, and obesity, which can lead to poor general health and a negative impact on HRQoL.^[43]^ This study suggests that seeking ways to reduce anxiety will help improve HRQoL even for people without chronic diseases.

The strength and significance of this study are in its comparisons between people with and without chronic diseases in terms of how factors such as depression, anxiety, and stress impact their HRQoL. However, this study also has limitations. First, we were unable to consider the severity of disease among chronically ill patients. Second, this study did not consider other factors that might influence HRQoL, including various psychological problems, social relationships, activities, and conditions such as eating disorders, psychological distress,^[44]^ personality disorders,^[45]^ and age differences between the 2 groups.^[46]^

## 5. Conclusion

This study compared factors affecting HRQoL between people with and without chronic diseases. Depression was found to have the greatest impact on the HRQoL of patients with chronic diseases, followed by exercise and economic status. Therefore, to improve HRQoL, it is necessary to diagnose depression in chronically ill patients early and actively implement interventions that can reduce it. To this end, more studies are needed on the relationship between depression, health-related behavior, and HRQoL, as well as on the development of systems for detecting depression early and exercise programs for chronic disease patients. Intervention studies focused on reducing anxiety should also be conducted to help improve the HRQoL of individuals without chronic diseases. Finding ways to resolve economic inequality will also improve HRQoL for all individuals.

## Author contributions

**Conceptualization:** Younghui Hwang, Jihyun Oh.

**Data curation:** Younghui Hwang, Jihyun Oh.

**Formal analysis:** Jihyun Oh.

**Funding acquisition:** Jihyun Oh.

**Investigation:** Younghui Hwang, Jihyun Oh.

**Methodology:** Younghui Hwang.

**Project administration:** Jihyun Oh.

**Resources:** Younghui Hwang.

**Software:** Younghui Hwang.

**Supervision:** Younghui Hwang.

**Validation:** Jihyun Oh.

**Writing – original draft:** Younghui Hwang, Jihyun Oh.

**Writing – review & editing:** Younghui Hwang, Jihyun Oh.
